# Clinico-radiologic subtypes and therapeutic observation of acute Marchiafava-Bignami disease

**DOI:** 10.1038/s41598-023-45431-6

**Published:** 2023-10-28

**Authors:** Yan-li Zhang, Chao Ran, Chao Xu, Wei Li

**Affiliations:** 1 Department of Clinical Pharmacy, Affiliated Hospital of Yangzhou University, Yangzhou, China; 2 Department of Radiology, Affiliated Yantai Yuhuangding Hospital of Qingdao University, Yantai, China; 3 Department of Medical Imaging, Affiliated Hospital of Yangzhou University, No. 368, Hanjiang Middle Road, Hanjiang District, Yangzhou, 225100 China

**Keywords:** Brain, Magnetic resonance imaging, Brain imaging, Neurotoxicity syndromes

## Abstract

We aimed to investigate the clinico-radiologic features of acute Marchiafava-Bignami disease (MBD) and its evolutionary process after effective treatment through subgroup comparison. The clinical and MRI data of 23 patients with acute MBD were retrospectively analyzed and divided into type A (12 cases, with entire callosal involvement) and type B (11 cases, with focal callosal involvement). The clinical assessments and MRI findings (before and after treatment) were compared between the two subtypes. Compared with type B, type A had lower MoCA (Montreal Cognitive Assessment) scores at admission (16.50 ± 1.73 vs 18.27 ± 1.68, P = 0.021) and were more common with extracallosal involvement (66.67% vs 18.18%, P = 0.036) and longer illness duration (18.3 ± 2.1 days vs 15.6 ± 2.4 days, P = 0.012). During the treatment, the residual lesion in the splenium was more common in type A (58.33% vs 9.09%, P = 0.027). After treatment, the MoCa scores of both subtypes gradually increased (P < 0.001), and the callosal and extracallasal lesions disappeared completely. Clinico-radiologic typing of acute MBD is related to the severity of early symptoms, but not to the prognosis. Complete clinico-radiologic recovery is possible for both subtypes with combined treatment. The clinico-radiologic reversibility is helpful for accurate diagnosis and therapeutic evaluation.

## Introduction

Marchiafava-Bignami disease (MBD) is a demyelinating lesion of the corpus callosum associated with alcoholism, which was first reported by Marchiafava and Bignami in 1903^1^. Its insidious acute onset results in non-specific clinical manifestations. Fortunately, MRI is an important imaging technique for accurate diagnosis, and DWI (diffusion-weighted imaging) can be used to monitor its therapeutic effects and evolutionary progress^2^. For a long time, acute MBD has been considered a fatal primary degeneration of the corpus callosum^3^. According to clinico-radiologic subtypes of Heinrich et al., compared with type B, extensive involvement and poor prognosis were more common in type A^4^. However, more and more cases of non-fatal acute MBD with good prognoses have been reported in recent years^5,6^. The potential reversibility of acute MBD has attracted much attention. Furthermore, there is no specific study to verify the clinico-radiologic subtypes of Heinrich et al. Therefore, 23 cases of acute MBD with regular treatment were collected in this study. Their MRI features and clinical assessments were analyzed to validate this clinico-radiologic typing and facilitate the accurate diagnosis and therapeutic evaluation.

## Materials and methods

### MRI examination and analyses

Twenty-three patients with acute MBD admitted to our hospital from Jan 2017 to July 2022 were included. By using 1.5 T or 3.0 T MR scanners (Philips Achieva, Eindhoven, Netherlands or GE Discovery MR750, Milwaukee, USA), T1-weighted imaging (T1WI), T2-weighted imaging (T2WI), fluid-attenuated inversion recovery (FLAIR) and diffusion-weighted imaging (DWI) sequences were performed for 23 patients. DWI was acquired by EPI sequence, b values = 0 and 1000 s/mm^2^.

Hyperintensity on DWI and hypointensity on the apparent diffusion coefficient (ADC) map were defined as restricted diffusion. The involved range of acute MBD was assessed on DWI. Measurements of ADC value were targeted at the splenium. The ADC value of each lesion was determined by an average value of three different regions of interest (ROIs, 10–30 mm^2^). The relative ADC (rADC) was used to obtain better comparability. Given that none of the 23 cases involved pons, the central part of pons was chosen as reference tissue. The ratio between the ADC values of callosal lesion and pons was defined as the rADC^7^. The distribution and signal characteristics of these lesions before and after treatment were reviewed separately by three experienced neuroradiologists, who were blind to the clinical diagnosis. When disagreements arose, a consensus was reached through consultation. According to the clinico-radiologic typing of Heinrich et al., type A is characterized by acute or subacute onset of the conscious disturbance, seizures, and pyramidal signs, with diffuse callosal involvement and poor prognosis. Type B is characterized by acute or subacute onset of cognitive impairment, dysarthria, and gait disturbance, with focal callosal involvement and a good prognosis^4^. Twenty-three cases were divided into type A or type B, and their imaging findings before and after treatment were compared.

### Clinical data and analyses

All patients were hospitalized for acute onset of symptoms such as conscious disturbance, cognitive impairment, dysarthria, ataxia, tetraparesis, behavioral changes, and delirium. They had a drinking history of 12–29 years, mainly Chinese liquor (40–50% proof), with an average daily intake of 200–250 ml. Each of them had a recent drunken experience before the first MRI examination (within 1–2 days). Their routine biochemical indexes (including routine blood and urine tests, liver and kidney function tests, and electrolyte tests) were all within the normal ranges.

After admission, methylprednisolone pulse therapy (500–1000 mg/day) and intravenous thiamine administration (500 mg/day) were performed for 3–5 days, followed by oral prednisone (60 mg/day, discretionary reduction) and compound B vitamins (3–9 tablets/day, each tablet contains Vitamin B1 3 mg, Vitamin B2 1.5 mg, Vitamin B6 0.2 mg, nicotinamide 10 mg, and calcium pantothenate 1 mg) for about 1–2 weeks. After discharge, all patients received alcohol withdrawal and oral compound B vitamins during follow-up (6–18 months). For both subtypes, Montreal Cognitive Assessment (MoCA) was applied for neuropsychological assessment at admission, discharge, and follow-up (2–3 months after discharge).

### Ethical considerations

This study was designed and conducted in accordance with the Declaration of Helsinki, and was approved by the Ethics Committee of Affiliated Yantai Yuhuangding Hospital of Qingdao University. Because it was a retrospective study, the Ethics Committee of Affiliated Yantai Yuhuangding Hospital of Qingdao University waived the requirement to obtain informed consent from the study participants.

### Statistical analysis

Descriptive statistics were performed to characterize clinico-radiologic features by IBM SPSS 22.0 (SPSS, Chicago, IL, USA). All data were presented as number (percentage) or mean ± SD. For subgroup comparison, an independent t-test (two-tailed) or Mann–Whitney U-test was used to assess the difference between continuous variables, and Fisher’s exact test (two-tailed) was used for dichotomous variables. The paired t-test was used to compare the MoCA scores of each subtype before and after treatment. P < 0.05 was defined as statistically significant. The inter-observer variability was examined by the Kappa factor. Kappa ≥ 0.8 was considered a better concordance.

## Results

### MRI findings and subtype comparisons

**Figure 1:**
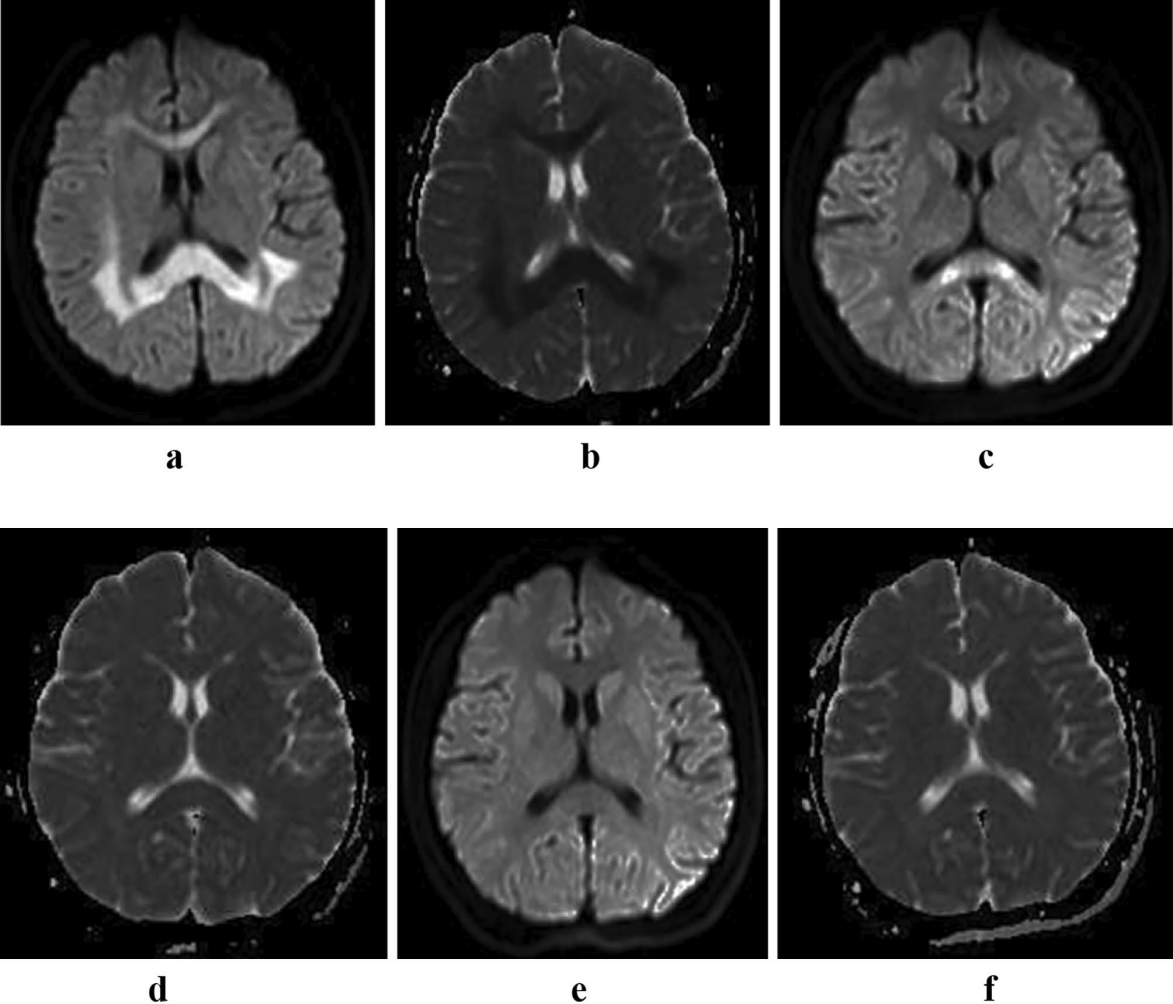
Type A acute MBD. Axial MRI scans showed extensive swelling of the corpus callosum with marked hyperintensity on DWI (**a**) and hypointensity on the apparent diffusion coefficient (ADC) map (**b**). Symmetrical restricted diffusion could be also observed in the bilateral hemispheric white matter (**a**,**b**). Six days after treatment, most intracerebral hyperintensity on DWI disappeared, except for the splenium (**c**), but it was unclear on the ADC map (**d**). Ten days later, the DWI hyperintensity in the splenium disappeared completely. Both DWI (**e**) and ADC map (**f**) were displayed normally.

**Figure 2:**
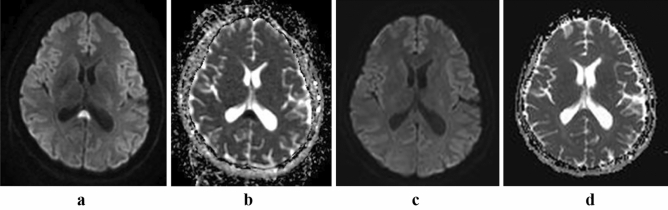
Type B acute MBD. Axial MRI scans displayed a focal lesion in the splenium, showing symmetrical hyperintensity on DWI (**a**) and hypointensity on the ADC map (**b**). Seven days after treatment, the lesion disappeared on the DWI (**c**) and ADC map (**d**).

A good inter-observer concordance was obtained for MRI analyses among the three independent observers (Kappa = 0.8396, P < 0.001). All 23 patients (12 cases of type A and 11 cases of type B) underwent MRI scanning before and after treatment, and the MRI was reexamined 1–2 times for clinical review. Before treatment, diffuse swelling of the corpus callosum was revealed in the 12 cases of type A, showing hyperintensity on DWI, and hypointensity on the ADC map. While the symmetric restricted diffusion in the splenium was seen in 11 cases of type B. There were no differences in rADC between these two subtypes. Compared with type B, the extracallosal involvements were more common in type A (66.67% vs 18.18%, P = 0.036), involving the bilateral hemispheric white matter. After treatment, the callosal and extracallosal restricted diffusion of both subtypes disappeared completely. Compared with type B, the residual DWI hyperintensity of splenium was more common in type A at the first reexamination (58.33% vs 9.09%, P = 0.027). In other words, the restricted diffusion disappeared earlier in the bilateral hemispheric white matter and anterior parts of the corpus callosum. See Figs. 1, 2, and Table 1 for details.

**Table 1:** Clinico-radiologic comparison between subtypes of acute MBD.

	Type A (n = 12)	Type B (n = 11)	P value
Age at onset (years)	44.8 ± 6.6	47.5 ± 6.0	0.317
Sex (male/female)	12/0	11/0	1.000
BMI (kg/m^2^)	26.87 ± 1.56	26.32 ± 1.35	0.380
Illness duration (days)	18.3 ± 2.1	15.6 ± 2.4	**0.012**
Familial history of alcoholism	5 (41.67%)	6 (54.55%)	0.537
Drinking history (years)	18.3 ± 5.2	21.5 ± 5.5	0.167
MoCA score (at admission)	16.50 ± 1.73	18.27 ± 1.68	**0.021**
MoCA score (at discharge)	26.17 ± 0.72	26.27 ± 0.79	0.740
MoCA score (at follow-up)	29.25 ± 0.75	29.36 ± 0.81	0.732
rADC	0.33 ± 0.26	0.31 ± 0.39	0.143
Extracallosal involvement	8 (66.67%)	2 (18.18%)	**0.036**
Residual lesion of splenium	7 (58.33%)	1 (9.09%)	**0.027**

Values are given as n (%) or mean ± SD.

*BMI* body mass index, *MoCA* montreal cognitive assessment, *rADC* relative apparent diffusion coefficient.

Significant values are in bold.

### Clinical findings and subtype comparisons

All 23 patients in this study were male, aged 36–56 years old (mean age 46.2 ± 6.3 years). The diagnosis of acute MBD was determined by the history of alcoholism, clinical manifestations, and MRI features. Both subtypes had similar ages, BMI (Body Mass Index), familial history of alcoholism, drinking history, and clinical symptoms. But the MoCA score of type A (16.50 ± 1.73) was lower than that of type B (18.27 ± 1.68, P = 0.021) at admission. Although type A had a longer illness duration (18.3 ± 2.1 days vs 15.6 ± 2.4 days, P = 0.012), the condition of all patients improved after treatment. At discharge, the MoCA score of each subtype (type A, 26.17 ± 0.72; type B, 26.27 ± 0.79) was higher than that at admission and reached normal levels (MoCA score ≥ 26, P < 0.001). During follow-up (2–3 months after discharge), the MoCA score of each subtype was further increased (type A, 29.25 ± 0.75; type B, 29.36 ± 0.81) compared with that at discharge (P < 0.001). However, there was no difference in MoCA score between the two subtypes at discharge and follow-up (P = 0.740 and P = 0.732). So far (6–18 months), no relapse or sequelae has been observed. See Table 1 for details.

## Discussion

Since both subtypes were essentially acute MBD, they had similar clinical characteristics and medical antecedents. However, type A lesions were extensive, resulting in a longer illness duration. The etiology and pathogenesis of acute MBD remain unclear. Nutritional and metabolic disorders caused by long-term alcohol consumption are considered to be its pathogenesis^8,9^. All 23 patients in this study were chronic alcoholics, recent drunkenness might be a predisposing factor for its acute onset. The direct neurotoxicity of alcohol can damage the callosal myelin^10^. MBD is one of the cytotoxic lesions of the corpus callosum (CLOCCs), which are a kind of secondary cytokinopathy^11^. Alcoholism can produce glutamate-mediated excitotoxicity on sodium–potassium pumps and aquaporins through cell-cytokine interactions^11,12^. Pathologically, it presents intramyelinic cytotoxic edema, myelin swelling, or demyelination^12,13^. DWI is the optimal sequence to detect cytotoxic damage, showing callosal restricted diffusion^14^.

Heinrich et al. proposed the clinico-radiologic subtypes of MBD in 2004, which provided a synthetic scale for its diagnosis and prognosis^4^. In this study, both subtypes had similar symptoms and rADC, indicating their common cytotoxic essence. However, the extent of restricted diffusion corresponded to the severity of symptoms. Type A showed diffuse callosal and extracallosal involvement with a lower MoCA score at admission. The corpus callosum connects the corresponding cortical areas through callosal radiation^15^. Bilateral hemispheric restricted diffusion may be the extracallosal involvement along the callosal radiation^16,17^. Callosal and extracallosal cytotoxic damage can compromise global neural interactions^18^.

For a long time, the extensive cytotoxic damage of MBD was considered irreversible, and with necrotic cavitation^3,14^. Both subtypes achieved complete clinico-radiologic recovery in this study, although type A had a lower MoCA score at admission. This indicated that acute MBD had potential reversibility, regardless of the extent of cytotoxic involvement. The rapid reversibility of these lesions suggested transient intramyelinic edema, rather than demyelination^19,20^. Because rapid remyelination is not possible^20,21^. In addition, these relatively young patients (mean age 46.2 ± 6.3 years) might have good resilience.

For type A, the DWI hyperintensity in the extra-callosum and anterior corpus callosum disappeared sooner than that in the splenium. It may be associated with the different degrees of damage. The splenium contains more glutamate excitatory receptors and is more susceptible to excitotoxicity^9,22^. Furthermore, it can be explained by the reversible splenial lesion syndrome (RESLES), which was proposed by Carcia-Monco et al. in 2011^23,24^. As a clinical-imaging syndrome, cytotoxic edema is its possible pathological basis^25^. RESLES can also be divided into two subtypes: RESLES I is more common, focally involving the splenium. RESLES II is rarer, extensively involving the callosal and extracallosal structures^26^. RESLES I and RESLES II may explain the longitudinal imaging changes of acute MBD. After effective treatment, the imaging patterns of type A were first changed from RESLES II to RESLES I. Eventually, all the lesions were undetectable. However, the investigations of RESLES have not yet reached a consensus.

Type A, especially with extracallosal involvement, indicates severe symptoms, slow recovery, and poor prognosis^4,27^. However, we found that the extent of restricted diffusion and MoCA score at admission did not affect the prognosis of acute MBD. The clinico-radiologic typing of Heinrich et al. was mainly based on the earlier literature review (1985–2003) and with a possible selection bias. Only relying on T2WI and CT to evaluate the callosal edema of acute MBD could bring about misjudgment and non-standardized outcomes. Early diagnosis and timely treatment can significantly improve the prognosis of acute MBD^20^. DWI can reveal the earlier lesions and wider distribution of acute MBD, and the combination of B vitamins and corticosteroids is effective for it^28,29^. Because B-vitamin deficiency can reduce neurotrophic and neurometabolic levels, the early supplement of high-dose thiamine is a neuroreparative treatment^30,31^. Continuous compound B vitamins oral administration is beneficial to nourishing nerves, inhibiting recurrence, and preventing complications^32^. For the direct neurotoxicity of alcohol, corticosteroid pulse therapy can alleviate brain edema, inhibit demyelination, and reduce inflammatory reactions^33^. Given the combination of corticosteroids and B vitamins, it is difficult to determine which one plays a major role in the treatment. However, the negative effects of combined treatment have not been reported.

Indeed, drunkenness might affect the initial MoCA score. However, its further elevation in the follow-up confirmed the continuous recovery of these patients. As a retrospective study, DWI acquired from different MR scanners cannot directly compare ADC values. rADC could reduce measurement bias to some extent. An invasive biopsy is not feasible for acute MBD. Therefore, multimodal imaging with a larger sample size is the best way to explore its pathogenesis.

Although all patients in this group had a clear history of alcohol consumption and a recent drunkenness, we could not rule out the possibility of other cytotoxic lesions of the corpus callosum. Drunkenness itself can also affect MoCA scores and confuse clinical evaluations at admission. Other advanced imaging methods were not available for this retrospective analysis, but DWI was sufficient to meet qualitative and quantitative needs. Due to the low incidence of acute MBD, the small sample size limited the statistical reliability. Further multi-modal imaging research with larger samples is necessary.

## Conclusion

Acute MBD is not always fatal, and complete recovery is possible. Early diagnosis and reasonable treatment are the key points. The clinico-radiologic typing of Heinrich et al. is not absolute for the evaluation of prognosis. The extent of callosal involvement is related to the severity of early symptoms, but not to the prognosis. The combination of B vitamins and corticosteroids is effective for both subtypes. The clinico-radiologic reversibility of acute MBD is helpful for clinical diagnosis and therapeutic evaluation.

## Data Availability

Data in this study are available from the corresponding author on reasonable request.
